# First description of atrial fibrillation and congenital thrombotic thrombocytopenic purpura treated by left atrial appendage occlusion

**DOI:** 10.1002/jha2.659

**Published:** 2023-03-03

**Authors:** Hussain Basrawala, Katherine Finley, Michael Jaglal, Bibhu D. Mohanty

**Affiliations:** ^1^ Department of Cardiovascular Sciences, University of South Florida Morsani College of Medicine Tampa Florida USA; ^2^ Department of Internal Medicine, University of South Florida Morsani College of Medicine Tampa Florida USA; ^3^ Department of Hematology and Oncology, University of South Florida Morsani College of Medicine Tampa Florida USA

**Keywords:** Anticoagulation, atrial fibrillation, LAAO, left atrial appendage, occlusion, purpura, stroke, thrombocytopenia, thrombocytopenic, thromboembolic, thrombosis, thrombotic, TTP, Watchman

## Abstract

Given an increased risk of both thrombosis and bleeding, thrombotic thrombocytopenic purpura (TTP) presents a unique challenge when anticoagulation is required for comorbid disease, particularly in the setting of major bleeding events. We present for the first time a patient with TTP and atrial fibrillation, presenting with recurrent stroke, but unable to tolerate anticoagulation due to prior intra‐cerebral hemorrhage. To address both issues concomitantly, we describe the successful application of a novel management approach to facilitate left atrial appendage occlusion, there by offering a non‐pharmacologic means of stroke prevention without added bleeding risk.

## 1 INTRODUCTION

Systemic anticoagulation (SAC) has long been the standard approach to thromboembolic (TE) stroke prevention in atrial fibrillation (AF). In patients with bleeding disorders, alternative approaches have been lacking, leaving patients to choose between heightened bleeding risk, or remaining unprotected from TE risk. Thrombotic thrombocytopenic purpura (TTP) presents a unique management conundrum when combined with AF. A thrombotic microangiopathy characterized by systemic microvascular platelet aggregation due to failure in degradation of von Willebrand factor (vWF) [1, 15], TTP can predispose patients to both thrombosis and hemorrhage due to marked thrombocytopenia – a relative contraindication to SAC.

Percutaneous left atrial appendage occlusion (LAAO) offers a non‐surgical means of treating stroke risk in AF by a local non‐pharmacologic approach, rather than the systemic standard. The left atrial appendage (LAA) contains corrugated pectinate muscle bands, which can harbor thrombus formed by blood stasis within a fibrillating left atrium, making this vestigial remnant responsible for >90% of thrombi yielding TE stroke related to AF [2]. Multiple clinical trials [3, 4, 5] have demonstrated the safety and efficacy of LAAO compared to SAC, and it is now routinely performed under a class 2 guideline recommendation [6]. Functionally, with LAAO occlusion, patients remain protected from stroke without requiring long‐term SAC. The standard LAAO protocol requires short‐term SAC (45 days post‐implantation) to ensure device healing and prevent thrombus formation on the newly implanted prosthesis.

Herein, we describe a patient with TTP, encumbered by considerable thrombotic and hemorrhagic morbidity, treated by a novel tailored LAAO approach to successfully offer clinical benefit while discontinuing SAC.

## 2 CASE

The patient is a 34‐year‐old male with congenital TTP, superior vena cava syndrome requiring stenting, multiple prior hospital admissions for transient ischemic attacks (TIA) and multi‐focal ischemic stroke, intracerebral hemorrhage (ICH), hypertension, hyperlipidemia and severe anemia with use of SAC. He was previously diagnosed with AF, with echocardiography confirming no valvular etiology. Given anemia, TTP, and ICH, he had been deemed a poor candidate for SAC.

**FIGURE 1:**
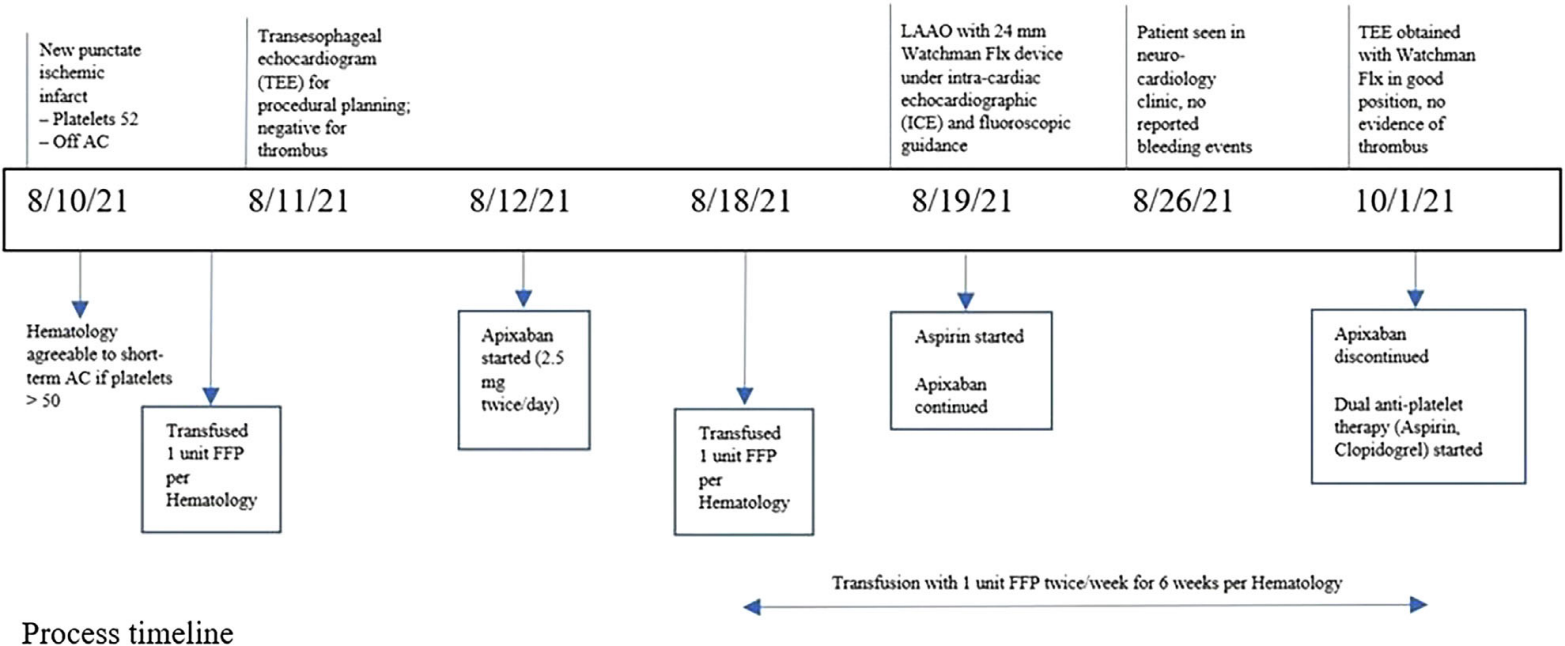
Process timeline.

In this instance, the patient presented with vision changes and expressive aphasia. Brain magnetic resonance imaging (MRI) and magnetic resonance angiography of the head/neck were unremarkable for acute insult and symptoms were attributed to TIA. Neuro‐vascular and Hematology services met to discuss preventative strategies. As long‐term anticoagulation was still felt to pose a high bleeding risk, the patient was referred to our Neuro‐cardiac service seeking non‐pharmacologic means to prevent stroke. LAAO was proposed if the patient could tolerate a short period of SAC. Hematology agreed to Apixaban use if the platelet count remained above 50,000/μl. However, given prior ICH, the patient elected discharge with plans to discuss in the outpatient setting. Unfortunately, he presented soon thereafter for sudden onset aphasia/dysarthria and asymmetric extremity weakness. He was ineligible for tissue plasminogen activator due to a platelet count of less than 100,000/μl. Brain MRI demonstrated a new infarct. After discussing LAAO in the face of recurrent ischemic events, the patient agreed to the following proposition (See Figure 1, Timeline): Half‐dose apixaban, taken 2.5 mg twice daily, would be initiated with anticipated implantation within a week. On the day of treatment initiation, trans‐esophageal echocardiography (TEE) completed for LAA sizing demonstrated normal left ventricular function and no significant valvular disease or intracardiac thrombi. The patient was discharged with no residual deficits from the acute infarct. During the intervening week, he was assessed by Hematology, was administered his standard fresh frozen plasma infusion to maintain platelet counts above 50,000/μl, and reported no bleeding or thrombotic events. Given the lack of thrombus on TEE and his ability to tolerate short‐term SAC, we proceeded with device implantation as planned. To permit rapid turnaround, and avoid endotracheal intubation and general anesthesia (which could mask neurologic effects), we offered to perform the procedure by a novel approach: under intra‐cardiac echocardiographic guidance and conscious sedation.

**FIGURE 2:**
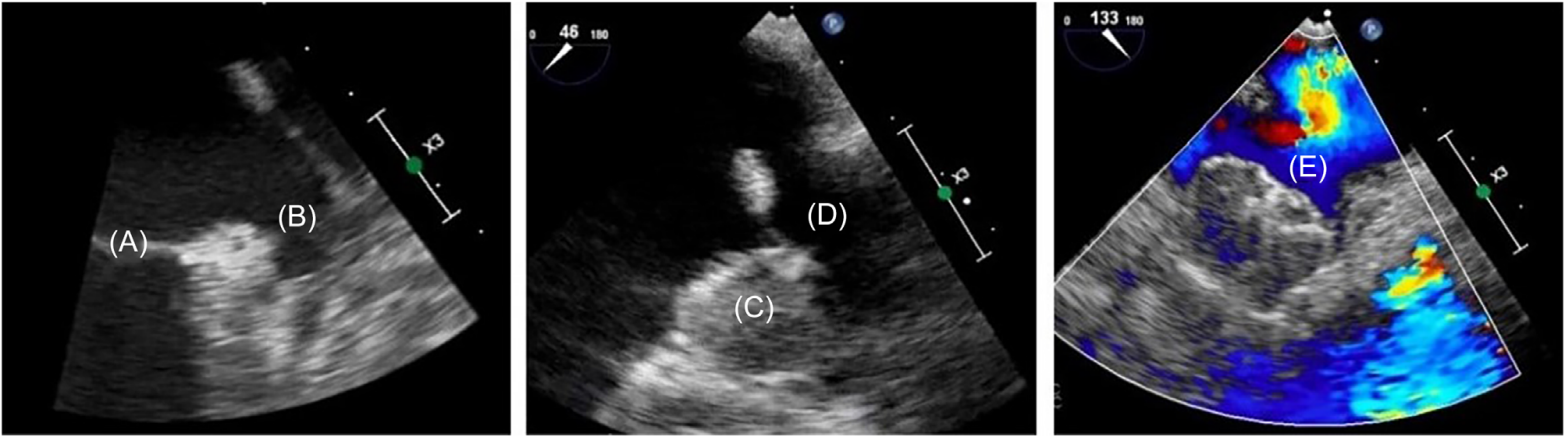
Transesophageal echocardiography: (A) Posterior leaflet of the mitral valve. (B) Left atrial appendage pre‐Watchman placement. (C) Left atrial appendage now with Watchman occluding the os. (D) Left upper pulmonary vein. (E) Color Doppler demonstrating no residual flow around the Watchman device.

A Watchman FLX device was successfully implanted without complications. He was discharged on apixaban 2.5 mg twice daily and aspirin 81 mg daily. At his post‐procedure visit with neuro‐cardiology, he denied any bleeding or repeat neurologic events. TEE at 45 days post‐implant demonstrated the LAAO device well‐positioned in the LAA with no evidence of device‐related thrombus or per‐device leak (See Figure 2). He was taken off apixaban and has not had any additional TE events over 1‐year follow‐up.

## 3 DISCUSSION

Advances in understanding congenital TTP have yielded a dramatic effect on prognosis. While untreated mortality is nearly 90%, the use of therapies such as plasma exchange has improved survival by nearly 85% [7, 8]. There remains a paucity of literature describing the management of congenital TTP patients with concomitant AF. The presence of thrombocytopenia poses a treatment dilemma due to the increased risk of both thromboembolism and bleeding events [9, 10]. Current AF guidelines offer little insight into the ideal management of patients on SAC with concomitant thrombocytopenia, although prospective studies have shown an increased incidence of mortality in this population [11]. This increased risk perhaps explains why patients with thrombocytopenia were largely excluded from previous randomized controlled trials (RTCs) evaluating the role of SAC in the prevention of TE stroke [12, 13]. Percutaneous LAAO has emerged as an alternative to long‐term SAC with similar efficacy in TE stroke prevention, but with a large reduction in bleeding risk. It is based on the observation that most thrombi, when seen by echocardiography in patients with nonvalvular AF, are located in the LAA [2]. The Watchman device has demonstrated efficacy in multiple RTCs, though standard implantation protocols mandate at least 45 days of post‐procedure SAC therapy to allow for endothelialization of the device. In the case of a well‐sealed device on TEE with minimal residual blood flow at 45 days, SAC can be discontinued, as seen in our patient, with continued long‐term low‐dose antiplatelet therapy [3].

To our knowledge, this is the first reported case of a patient with TTP and AF successfully treated with LAAO for the prevention of TE stroke. We also applied a novel, tailored process by which this patient could be successfully treated, with contingencies incorporated to allow for clinical variability. While there are limited small retrospective studies showing that LAAO may be an efficacious and safe option for patients with thrombocytopenia, there is still a need for larger trials assessing the long‐term implications of LAAO in AF patients with concurrent thrombocytopenia [14].

## CONFLICT OF INTEREST STATEMENT

Bibhu D. Mohanty serves on the medical advisory board and speaker's bureau for Boston Scientific is on the speaker's bureau for Abbott and has received consulting fees and grant support from Medtronic, Edwards Life Sciences, and W. L. Gore and Associates. The remaining authors have no conflict of interest, financial or otherwise, to disclose.

## FUNDING INFORMATION

The authors received no specific funding for this work.

## ETHICS STATEMENT

N/A

## Data Availability

Data sharing is not applicable to this article as no new data were created or analyzed in this study.
